# Psychosocial factors associated with persistent pain in people with HIV: a systematic review with meta-analysis

**DOI:** 10.1097/j.pain.0000000000001369

**Published:** 2018-08-16

**Authors:** Whitney Scott, Chinar Arkuter, Kitty Kioskli, Harriet Kemp, Lance M. McCracken, Andrew S.C. Rice, Amanda C. de C. Williams

**Affiliations:** a Health Psychology Section, Institute of Psychiatry, Psychology, and Neuroscience, King's College London, London, United Kingdom; b Pain Research Group, Department of Surgery and Cancer, Faculty of Medicine, Imperial College London, London, United Kingdom; c INPUT Pain Management Unit, Guy's and St Thomas' NHS Foundation Trust, London, United Kingdom; d Research Department of Clinical, Educational, and Health Psychology, University College London, London, United Kingdom

**Keywords:** HIV, Pain, Systematic review, Psychosocial factors

## Abstract

Chronic pain remains a prevalent and disabling problem for people living with HIV in the current antiretroviral treatment era. Psychosocial treatments may have promise for managing the impact of this pain. However, research is needed to identify psychosocial processes to target through such treatments. The current systematic review and meta-analysis examined the evidence for psychosocial factors associated with pain, disability, and quality of life in people living with HIV and persistent pain. Observational and experimental studies reporting on the association between one or more psychosocial factors and one or more pain-related variables in an adult sample of people living with HIV and pain were eligible. Two reviewers independently conducted eligibility screening, data extraction, and quality assessment. Forty-six studies were included in the review and 37 of these provided data for meta-analyses (12,493 participants). “Some” or “moderate” evidence supported an association between pain outcomes in people with HIV and the following psychosocial factors: depression, psychological distress, posttraumatic stress, drug abuse, sleep disturbance, reduced antiretroviral adherence, health care use, missed HIV clinic visits, unemployment, and protective psychological factors. Surprisingly, few studies examined protective psychological factors or social processes, such as stigma. There were few high-quality studies. These findings can inform future research and psychosocial treatment development in this area. Greater theoretical and empirical focus is needed to examine the role of protective factors and social processes on pain outcomes in this context. The review protocol was registered with PROSPERO (CRD42016036329).

## Abstract 2

Supplemental Digital Content is Available in the Text.

## 1. Introduction

HIV remains a significant global health concern with 36.7 million people living with HIV worldwide.^130^ The availability of combined antiretroviral therapy (cART) has drastically improved life expectancy.^9,93,120^ In well-resourced countries, and increasingly in less well-resourced regions, the shift in HIV from a terminal illness to a chronic condition has led to a focus on disease and symptom management.^59^

Chronic pain is a common symptom in people with HIV. Data from one systematic review indicate that 54% to 83% of people with HIV may experience clinically meaningful persistent pain, and these estimates seem to be stable from the pre- to current-cART era.^80^ Neuropathic pain is a frequent complication of HIV and/or antiretroviral therapy.^15^ Approximately 42% to 66% of people with HIV have peripheral sensory neuropathy (HIV-SN), and around 54% to 78% of these experience neuropathic pain.^84,88,129^ Importantly, pain in people with HIV is associated with increased disability and reduced quality of life.^27^

There are few pharmacological options for managing chronic HIV-related pain. A systematic review of 14 randomized controlled trials (RCTs) of pharmacotherapy for painful HIV-SN found efficacy only for topical capsaicin, smoked cannabis, and subcutaneous nerve growth factor.^85^ However, nerve growth factor is not clinically available, capsaicin is not feasible in lower-resourced settings, and a subsequent review of cannabis showed no effect on neuropathic pain and concerns about long-term side effects.^32^ Additional negative RCTs of pregabalin, capsaicin, and amitriptyline have been published.^17,24,103^

In the wider literature, psychological approaches are common in chronic pain management.^34^ Psychological treatments, including cognitive–behavioural therapy (CBT), are associated with improved functioning and mood for chronic pain that is primarily musculoskeletal.^124^ However, research on psychological treatments for pain in HIV is less well developed. Only 2 RCTs have examined CBT for people with HIV and chronic pain, but interpretation of these trials is hampered by small samples^118^ and high dropout rates.^29^ An observational study of CBT for HIV-related pain showed similarly poor treatment completion.^21,113^ There is a clear need for improving psychological treatments for people with HIV-related pain.

Improving psychological approaches for chronic pain in HIV will require consideration of the psychosocial complexities associated with HIV. For example, stigma, mental health problems, and substance abuse may influence pain and treatment engagement in people with HIV.^36,69,71,111,123,132^ However, research has not systematically examined psychosocial factors associated with pain in this context. The systematic review by Parker et al. (2014), which estimated the prevalence of pain in HIV, described 5 studies reporting psychosocial factors. However, that review did not specifically include assessment of psychosocial factors in the eligibility criteria. Furthermore, 33 potentially eligible studies were excluded due to low-quality ratings,^80^ which limits our understanding of the range of psychosocial factors examined in this context. Therefore, we conducted a systematic review and meta-analyses to examine the associations between psychosocial factors and persistent pain in HIV. Because the aims of the review were exploratory, we did not formulate specific hypotheses about the associations between these variables.

## 2. Methods

The review protocol was registered with PROSPERO (http://www.crd.york.ac.uk/PROSPERO/display_record.php?ID=CRD42016036329).

### 2.1. Inclusion and exclusion criteria

#### 2.1.1. Inclusion

(1) People with HIV aged 18 years and older.
(2) The original protocol specified the study must have a “(sub)sample with average pain duration of ≥3 months.” After piloting this criterion, a large number of studies did not define or report pain duration. We contacted authors to enquire about pain chronicity; however, these data were generally not available. Given the potentially high prevalence of chronic pain in HIV,^80^ we decided to include studies with (sub)samples of ambiguous pain duration, provided that “pain” vs “no pain” subgroup analyses were reported in studies for which chronic pain was not an eligibility criterion or which did not report pain duration.
(3) Data on presence of pain, pain intensity, functioning, and/or quality of life.
(4) Data on one or more psychosocial variable, representing any potentially modifiable cognitive, affective, behavioural, or interpersonal process. Adherence to antiretroviral therapy and health care use variables represent modifiable behaviour patterns. Therefore, we considered these as psychosocial variables eligible for this review.
(5) Observational (cross-sectional, case-control, or prospective) or experimental studies (RCTs) reporting between- or within-groups associations between pain and psychosocial variables in a (sub)sample with pain.
(6) Any language, from any region, from 1981 onwards (the date that HIV was identified in the literature).
(7) Studies (published and unpublished) with an available full-text. Where only abstracts or trial registration summaries were available, the authors provided unpublished data for the review. Unpublished studies are commonly included in systematic reviews, given recognition of overestimation of effects in published research.^62^ In addition, studies conducted in lower-resourced countries where HIV is particularly prevalent may not always proceed through to publication. Therefore, the inclusion of unpublished studies and dissertations may help overcome this disparity and allows us to consider potential contextual differences.

#### 2.1.2. Exclusion

(1) Studies only measuring associations between unchangeable demographic factors (eg, age, ethnicity) and pain. While piloting the eligibility criteria, we found a number of studies reporting history of injecting drug use as participants' HIV risk factor. Given lack of further information about substance abuse history or current abuse, we excluded studies for which injecting drug use history was the only psychosocial factor. Likewise, we excluded studies reporting only average units of alcohol consumed, rather than alcohol abuse.
(2) Qualitative studies.

### 2.2. Search strategy

We searched the following databases during March 2016: Medline, EMBASE, CINAHL, PsycINFO, Cochrane, and Web of Science. We also searched ISRCTN, clinicaltrials.gov, and EU Clinical Trials Register. Reference lists of eligible studies were searched and key authors were contacted. We reran the search in August 2017. The search included terms for the target population (HIV or AIDS), outcome (chronic pain), and exposure measurement (psychosocial factors). Relevant search terms were identified from previous reviews on pain in HIV,^80,124^ psychosocial factors in HIV,^99^ and psychosocial factors in chronic pain^40^ (Appendix A, available at http://links.lww.com/PAIN/A643).

### 2.3. Data extraction

Two reviewers (W.S. and C.A.) independently screened titles/abstracts and full-texts for eligibility. The following data were extracted from eligible studies: year; design; country; sample size; demographics (ie, age, sex, and race/ethnicity); clinical factors (ie, HIV duration, use of ART, CD4^+^ count and viral load, and pain duration and type); assessment of pain and psychosocial variables; and, statistical analyses. In cases where both cross-sectional and prospective data reported the same (or an overlapping) cohort and variables, the prospective analyses were extracted. Data were extracted from all studies by W.S., and independently by C.A. and K.K. who each extracted data from approximately half of the studies. Disagreements regarding eligibility and data extraction were discussed to reach consensus and, where discrepancies remained, W.S. discussed these with the wider team. The reviewers were not blinded to the authorship of the studies reviewed.

### 2.4. Quality assessment

We assessed the methodological quality of studies using an adapted version of quality assessment tools used in previous systematic reviews of observational studies relevant to pain and HIV.^3,40,80^ The quality assessment tool contained items assessing: study purpose, recruitment, response rates, sample description, assessment measurements, data analysis, and confounding/matching (Appendix B, available at http://links.lww.com/PAIN/A643). Additional items assessed features specific to prospective designs. Thus, quality scores differed for cross-sectional and prospective studies. In some cases, the overall study design did not correspond to the nature of the data extracted for the purpose of this review. In addition, in some cohorts, a cross-sectional design was used to examine one psychosocial variable, whereas a prospective design was used to examine another variable using the same sample. In all cases, the quality assessment was applied to the design used for the nature of the data extracted for a given psychosocial variable. Quality assessment items were rated as “positive” (1), “negative” (0), or “unclear” (?), and total scores were computed and classified as low (<50%), medium (50%-80%), and high (>80%).^3,40^ W.S. completed quality assessment ratings for all studies, whereas C.A. and K.K. each independently completed the quality assessment for approximately half of the studies. The strength of evidence was assessed according to the levels outlined by Ariëns et al.^3^ in a systematic review of observational studies of psychosocial risk factors for neck pain: (1) Strong: consistent results in multiple high-quality prospective and/or case-control studies; (2) Moderate: consistent results in multiple prospective and/or case-control studies; (3) Some Evidence: findings in one prospective or case-control study, or consistent findings in multiple cross-sectional studies with at least one high-quality study; and (4) Inconclusive: Inconsistent findings in multiple studies or consistent findings in multiple low-quality cross-sectional studies.^3^

### 2.5. Data synthesis

Meta-analyses were conducted using Stata 15.0 where there were at least 2 studies^43^ with the same design and effect estimate of the association between the same pain (eg, intensity) and psychosocial variables (eg, depression). We took a broad approach to the meta-analyses,^37,45^ and grouped psychosocial variables on the basis that they reflected conceptually similar underlying constructs with overlapping measurement content. All analyses were conducted using random effects, given likely heterogeneity.^43^ Between-study heterogeneity (I^2^ statistic) was interpreted as low (<25%), medium (25%-50%), and high (>50%).^124^ For between-groups comparisons of continuous data, mean values, SDs, and sample sizes were extracted to compute the pooled standardized mean difference (SMD). For between-groups comparisons of dichotomous data, events data and sample sizes were extracted. Where events data were not reported, odds or hazard ratios, 95% confidence intervals (95% CIs), and sample sizes were extracted. To aggregate studies reporting a mixture of odds ratios (ORs) and events data, ORs were first computed from studies reporting events data and then pooled with ORs reported in other studies. Odds ratios and hazard ratios were analysed separately. Where applicable, correlation coefficients (Pearson *r*) were extracted with sample sizes. We transformed *r* to Fisher *z* and computed 95% CIs of *z* to compute the pooled estimate.^18,100^

Data extracted were from bivariate analyses. Multivariate data (eg, adjusted ORs) were only extracted where bivariate data were not available. We focused on bivariate analyses because many studies did not report multivariate analyses. Moreover, studies that reported multivariate models varied substantially with respect to control variables included and inconsistently used psychosocial variables as independent or dependent variables. Taken together, these differences limit meaningful interpretation of multivariate analyses across studies.

Several studies presented data on more than 2 pain/no pain groups, often with idiosyncratic group definitions, which limited our ability to compare studies. Where studies reported 3 or more groups, we collapsed these into 2 to represent groups with and without pain (eg, frequent/moderate/severe vs infrequent/mild/none), and computed effects between these. This approach facilitated more direct comparison across studies and thus enabled us to include a larger number of studies in the analyses. For studies comparing participants on the presence of neuropathy, we prioritised extracting data from these comparisons in the following order depending on the data reported: (1) painful vs nonpainful neuropathy; (2) painful neuropathy vs no neuropathy; and (3) neuropathy vs no neuropathy. Where there was more than one measure of the same variable, we extracted data for the measure with the widest usage or the longer measure to increase reliability.^124^ Our protocol specified that funnel plots would be inspected to assess for publication bias. However, due to the relatively small number of studies in each meta-analysis and the likelihood of high heterogeneity, inspection of funnels plots was not appropriate^109^ and, therefore, was not undertaken.

We conducted sensitivity analyses to examine the influence of the following study and patient characteristics on the findings: certainty of pain chronicity, pain type, immune functioning and viral suppression, ART treatment era, and health care system. With the exception of the pain chronicity analysis, these sensitivity analyses were prespecified. Given the large number of potential analyses, we restricted sensitivity analyses to the between-groups SMDs for depression because this was the analysis with the largest number of studies.

## 3. Results

**Figure 1.**
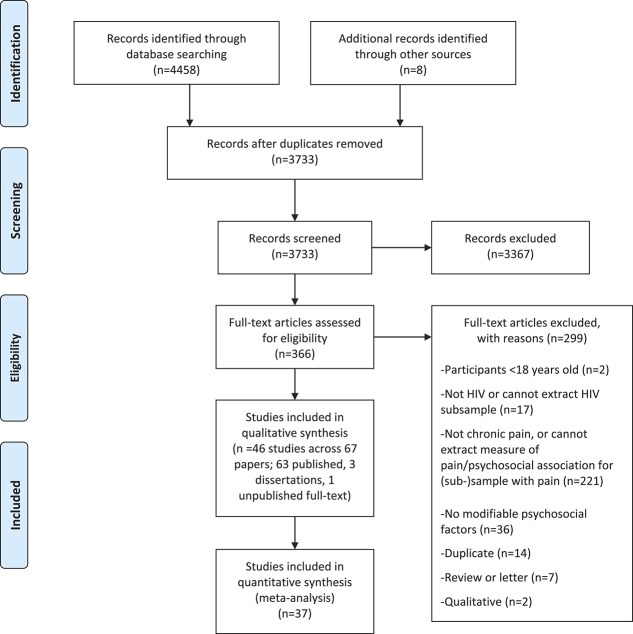
Preferred Reporting Items for Systematic Reviews and Meta-Analyses (PRISMA^75^) flow diagram.

Forty-six studies were included in the review (13,480 participants) and 37 of these provided data for meta-analyses (12,493 participants; Fig. 1)

. Most (83%) were conducted in the United States, with 4 studies from South Africa,^79,88,121,122^ and one each from the United Kingdom,^84^ Thailand,^89^ Uganda,^95^ and Russia.^114^ Participants were primarily recruited from HIV clinics or using multifaceted strategies that also included recruitment from substance abuse clinics and community outreach. One study recruited exclusively from a methadone clinic,^8^ whereas 2 others recruited in high-poverty areas.^39,110^ The samples comprised predominantly men in 41 studies, with the proportion of men in these studies ranging from 51%^50^ to 100%.^28,104^ Five studies (4 from South Africa and 1 from the United States) recruited women exclusively^79,92^ or predominantly (proportion of women ranging from 72% to 88%).^88,121,122^ The mean age ranged from 30.1 (SD = 5.2)^114,115^ to 51.0 (SD = 9.3) years.^119^ HIV duration was not consistently reported; however, of the studies providing data, duration ranged from 2.09 (SD = 1.22)^102^ to 16.95 years (SD = 8.70).^119^ Eighteen studies (39%) reported on mixed HIV/AIDS samples (reported proportion with AIDS ranged from 10% to 74%). Four studies included only participants with AIDS, one study excluded patients with AIDS, whereas 23 studies did not clearly report the proportion (if any) with AIDS. Supplemental Table 1 shows further demographic characteristics of the study samples (available at http://links.lww.com/PAIN/A643).

Table 1 provides a summary of study designs, quality, and evidence level for each psychosocial factor. The studies showed substantial variability in the measurement of pain and psychosocial variables. Most studies (63%) were of medium quality. Fifteen studies were of low quality, and only 2 were of high quality.^79,84^ The most common limitations included unclear reporting of response rates, no a priori sample size justification, and poor reporting of HIV and pain characteristics. There is no single agreed upon strategy to best address low-quality studies within meta-analyses, an issue which is compounded by the arbitrary nature of study quality scoring and cutoff points.^43^ This can be dealt with by only including high-quality studies, performing sensitivity analyses, or including all studies irrespective of quality and discussing risk of bias.^43^ Given that only 2 of 46 studies were rated as high quality, a meta-analysis of these cannot be regarded as reflecting most of the studies. Sensitivity analyses would likewise not be meaningful. Including all studies is thus the most justifiable approach for the current data. Although we have chosen to focus on data from bivariate analyses for reasons outlined in the Methods, studies that reported a multivariate model of the association between psychosocial and pain variables are shown in bold in Table 1 for ease of reference.

**Table 1:**
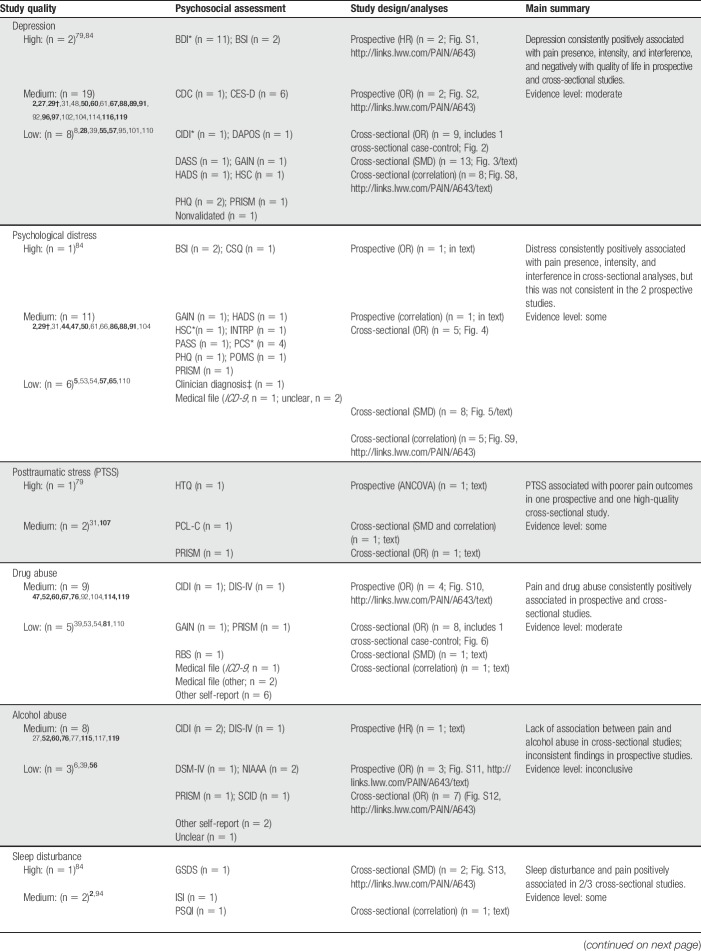

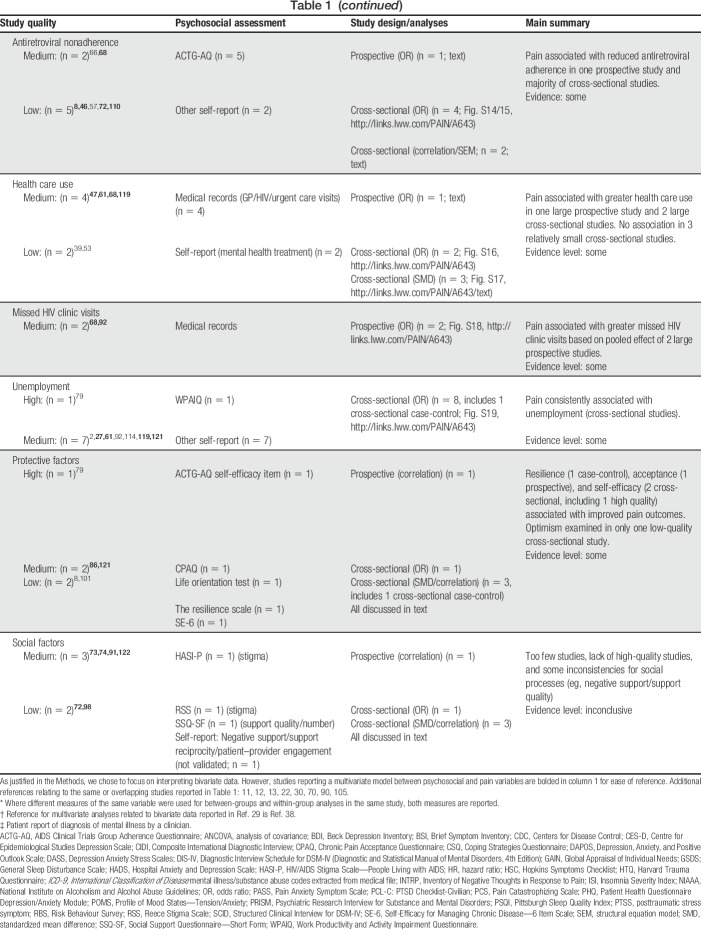
Summary of the evidence for psychosocial variables.

### 3.1. Depression

**Figure 2.**
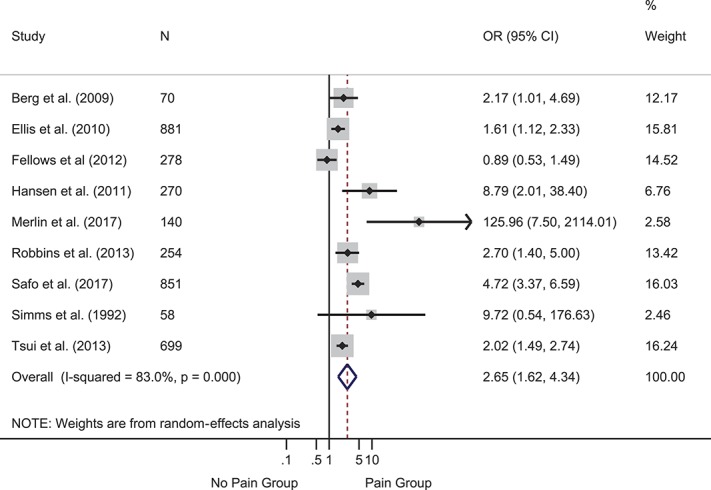
Forest plot of cross-sectional odds ratios (ORs) for depression. Depression was more likely in participants with vs without pain, as reflected in the pooled OR of >1. Gray boxes show weighting of individual studies; the red dotted line indicates the pooled effect around which effects from individual studies vary; the blue diamond shows the 95% CI around the pooled effect. CI, confidence interval.

**Figure 3.**
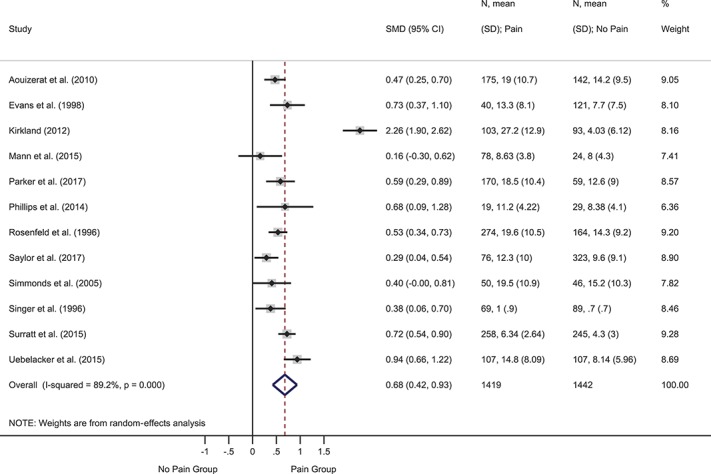
Forest plot of cross-sectional standardized mean differences (SMDs) for depression. Depression symptoms were more severe in participants with vs without pain, as indicated by a positive pooled SMD. CI, confidence interval.

Depression was the most frequently assessed psychological variable, investigated in 29 studies. Two prospective studies reported hazard ratios for baseline depression predicting time to onset of symptomatic neuropathy. The pooled hazard ratio was significant and indicated that baseline depression was more severe in participants who developed symptomatic neuropathy at follow-up than those who did not: HR = 1.04 (95% CI 1.02-1.07), *z* = 3.23, *P* = 0.001 (supplemental Figure 1, available at http://links.lww.com/PAIN/A643). Heterogeneity was 0.0%. Two further prospective studies reported ORs. The pooled OR was significant and indicted that higher baseline depression symptoms were associated with greater likelihood of follow-up pain: OR = 2.26 (95% CI 1.47-3.47), *z* = 3.72, *P* < 0.001 (supplemental Figure 2, available at http://links.lww.com/PAIN/A643). Heterogeneity was medium (40.1%). Nine cross-sectional studies provided events data or ORs. The pooled OR was significant such that depression was more likely in participants with vs without pain: OR = 2.65 (95% CI 1.62-4.34), *z* = 3.90, *P* < 0.001 (Fig. 2). Heterogeneity was high (83.0%). Twelve cross-sectional studies provided data to compute SMDs (Fig. 3). The overall effect was significant and showed moderately greater depression in participants with vs without pain: SMD = 0.68 (95% CI 0.42-0.93), *z* = 5.22, *P* < 0.001. Heterogeneity was high (I^2^ = 89.2%). Another cross-sectional study that reported the median and interquartile range found no difference in depression between groups with (n = 125) and without pain (n = 72).^88^

Six cross-sectional studies reported correlation coefficients between depression and pain severity. The pooled correlation was small, but significant: Fisher *z* = 0.26 (95% CI 0.18-0.33), *z* = 6.77, *P* < 0.001. Heterogeneity was 0.0%. One additional study found a nonsignificant correlation, although the coefficient was not reported.^27^ Four cross-sectional studies reported correlations between depression and pain interference/disability. The pooled correlation was moderate: Fisher *z* = 0.48 (95% CI 0.41-0.56), *z* = 12.48, *P* < 0.001. Heterogeneity was 0.0%. Three cross-sectional studies reported correlations between depression and quality of life. The pooled correlation was large and significant: Fisher *z* = −0.52 (95% CI −0.75 to −0.30), *z* = 4.51, *P* < 0.001. Heterogeneity was high at 73.3% (all correlation analyses, supplemental Figure 8, available at http://links.lww.com/PAIN/A643). One final cross-sectional study (n = 120) reported a moderate correlation between pain presence and depression.^55^

### 3.2. Depression sensitivity analyses

We conducted sensitivity analyses on the SMDs for depression between pain and no pain groups (supplemental Figures 3–7, available at http://links.lww.com/PAIN/A643). We excluded data from the Kirkland study here because the SMD from this study was substantial and seemed to be driving heterogeneity in the primary meta-analysis. Excluding the Kirkland data reduced heterogeneity from I^2^ = 89.2% to 52.2%. Thus, 11 studies were included in sensitivity analyses.

The pooled SMD for depression was medium for studies with certain (0.61, 95% CI 0.11-1.12, *z* = 2.39, *P* = 0.02; I^2^ = 75.1%) and uncertain pain chronicity (0.53, 95% CI 0.41-0.64, *z* = 9.04, *P* < 0.001; I^2^ = 34.5%). In the analysis by pain type, the pooled SMD for depression was moderate in studies with mixed pain types (0.75, 95% CI 0.58-0.92, *z* = 8.51, *P* < 0.001; I^2^ = 31.7%) and for which pain type was not reported (0.52, 95% CI 0.39-0.65; *z* = 7.91, *P* < 0.001; I^2^ = 0.0%). By contrast, studies with neuropathic pain (0.31, 95% CI 0.11-0.52; *z* = 2.96, *P* = 0.003; I^2^ = 0.0%) or headache (0.38, 95% CI 0.06-0.70, *z* = 2.34, *P* = 0.02) showed small but significant differences between groups on depression.

The pooled SMD for depression was moderate for studies in which participants had less than adequate immune functioning and viral suppression (0.56, 95% CI 0.42-0.70, *z* = 7.77, *P* < 0.001; I^2^ = 0.0%) and for studies in which these indicators were uncertain (0.53, 95% CI 0.33-0.73, *z* = 5.28, *P* < 0.001; I^2^ = 69.1%). There were no studies with “adequate” functioning on these indices in this analysis. Studies from the pre-cART (0.49, 95% CI 0.32-0.66, *z* = 5.75, *P* < 0.001; I^2^ = 0.0%), cART (0.73, 95% CI 0.37-1.10, *z* = 3.91, *P* < 0.001), and current-cART era (0.55, 95% CI 0.38-0.72, *z* = 6.21, *P* < 0.001; I^2^ = 62.5%) all had moderate or near-moderate pooled SMDs. Finally, pooled SMDs for depression were similar in studies from the United States, which has a mixed health care system (0.57, 95% CI 0.43-0.72, *z* = 7.58, *P* < 0.001; I^2^ = 55.7%), and one study from the United Kingdom, which has universal health care (0.68, 95% CI 0.09-1.28; *z* = 2.25, *P* = 0.03). The pooled SMD of 2 studies conducted in lower- and middle-income countries was smaller, but statistically significant (0.43, 95% CI 0.14-0.72, *z* = 2.90, *P* = 0.004; I^2^ = 54.2%).

### 3.3. Psychological distress

**Figure 4.**
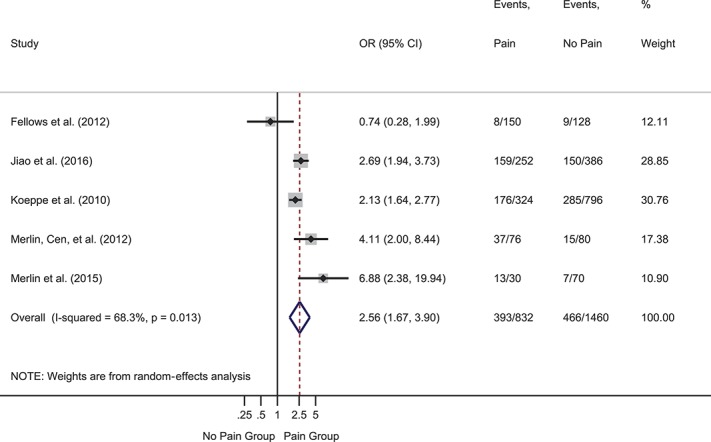
Forest plot of cross-sectional events data for psychological distress. Distress was more likely in participants with vs without pain, as reflected in the pooled odds ratio (OR) of >1. CI, confidence interval.

**Figure 5.**
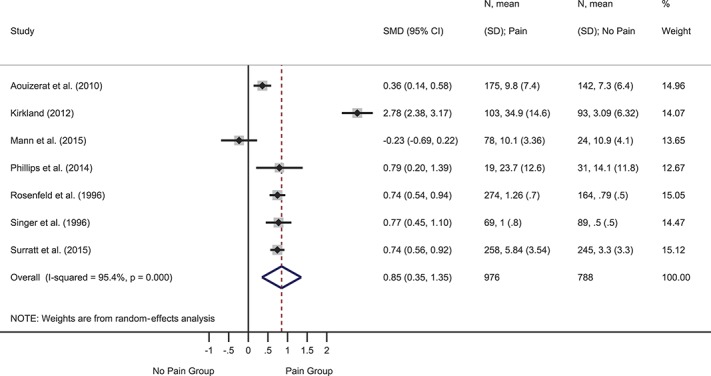
Forest plot of cross-sectional standardized mean differences (SMDs) for psychological distress. Distress was more severe in participants with vs without pain, as reflected by a positive pooled SMD. CI, confidence interval.

Eighteen studies examined variables representing psychological distress, including anxiety-related constructs and the presence of “mental illness,” which generally described a combination of anxiety and depression. Five cross-sectional studies provided events data (Fig. 4). The pooled OR was significant and indicated that participants with pain were more likely to have psychological distress than those without pain: OR = 2.56 (95% CI 1.67-3.90), *z* = 4.34, *P* < 0.001. Heterogeneity was high (I^2^ = 68.3%). One prospective study (n = 127) found that baseline mental illness did not predict presence of pain over follow-up.^54^ Seven cross-sectional studies provided mean values and SDs (Fig. 5). The pooled SMD showed a large and statistically significant difference between groups such that distress was worse in participants with vs without pain (SMD = 0.85, 95% CI 0.35-1.35); *z* = 3.33, *P* = 0.001). Heterogeneity was very high (I^2^ = 95.4%). One further study that reported the median and interquartile range found no difference between groups.^88^

Four cross-sectional studies reported correlations between distress and pain severity (supplemental Figure 9, available at http://links.lww.com/PAIN/A643). The pooled correlation was moderate: Fisher *z* = 0.35 (95% CI 0.09-0.60), *z* = 2.68, *P* = 0.007. Heterogeneity was high (82.2%). Three cross-sectional studies reported correlations between distress and pain interference/disability. The pooled correlation was moderate: Fisher *z* = 0.59 (95% CI 0.24-0.93), *z* = 3.34, *P* = 0.001. Heterogeneity was high (81.2%). One prospective study (n = 45-62) found a nonsignificant correlation between change in distress and pain severity after CBT, and a significant, moderate correlation between change in distress and pain interference.^44,86^ Finally, one cross-sectional study reported a nonsignificant correlation between pain intensity and distress (*r* not reported), and a small negative correlation between distress and quality of life.^88^

### 3.4. Posttraumatic stress

Three studies investigated posttraumatic stress. These studies are reported separate from studies measuring psychological distress, given the specificity of posttraumatic stress as a variable. Different study designs and analyses precluded meta-analysis. One prospective study (n = 143) found that posttraumatic stress symptoms (PTSSs) were associated with significantly higher pain severity and interference over time in a sample with HIV and persistent pain.^107^ One high-quality cross-sectional study found that participants with pain (n = 170) had significantly higher PTSS than those without pain (n = 59).^79^ Within the pain group in this study, there was a nonsignificant correlation between PTSSs and pain severity, and small but significant correlations between PTSSs and pain interference (positive correlation) and quality of life (negative correlation).^79^ Posttraumatic stress disorder did not differ between groups with (n = 150) and without (n = 128) neuropathy in another cross-sectional study.^31^

### 3.5. Drug abuse

Fourteen studies examined drug abuse. We prioritised extracting opioid abuse data when multiple drug abuse categories were reported, given the relevance of opioid use in chronic pain. Two prospective studies reported ORs for pain predicting heroin use at the time of follow-up. The pooled OR indicated that participants with pain at baseline were more likely at follow-up to be using heroin: OR = 1.70 (95% CI 1.22-2.38), *z* = 3.13, *P* = 0.002 (supplemental Figure 10, available at http://links.lww.com/PAIN/A643). Heterogeneity was low (I^2^ = 14.0%). Conversely, another prospective study (n = 493) reported that baseline opioid use disorder history predicted new onset of neuropathic pain, OR = 2.87 (1.31-6.28), *P* < 0.01.^60^ One low-quality prospective study (n = 127) found that baseline drug abuse history did not predict the presence of pain at follow-up, 0.55 (0.25-1.21).^54^ These 2 studies could not be combined due to different coding of the dependent variable.

**Figure 6.**
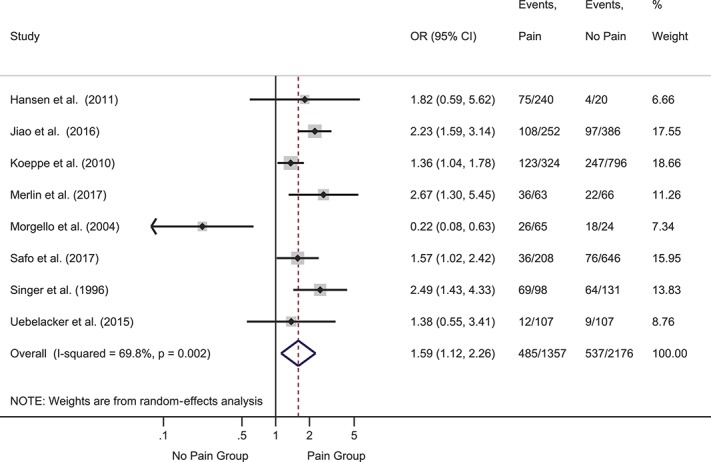
Forest plot of cross-sectional events data for drug abuse. Drug abuse was more likely in participants with vs without pain, as reflected in the pooled odds ratio (OR) of >1. CI, confidence interval.

Eight cross-sectional studies reported events data (Fig. 6). The pooled OR was significant such that participants with pain were more likely to have comorbid drug abuse than those without pain: OR = 1.59 (95% CI 1.12-2.26), *z* = 2.58, *P* = 0.01. Heterogeneity was high (I^2^ = 69.8%), mainly attributable to one study that found the opposite effect, such that participants with symptomatic distal sensory polyneuropathy were less likely to have opioid use disorder than those with asymptomatic distal sensory polyneuropathy.^76^ One low-quality cross-sectional study (n = 503) found that participants with “untreated” pain had greater dependence symptoms than those with “treated” pain or without pain.^110^ Another low-quality cross-sectional study (n = 73) found a small positive correlation between “aberrant drug behaviours” and pain interference, but not quality of life.^81^

### 3.6. Alcohol abuse

Eleven studies investigated alcohol abuse. Two prospective studies reported ORs for baseline pain predicting subsequent alcohol abuse. The pooled OR was not significant: OR = 0.94 (95% CI 0.39-2.26), *z* = 0.13, *P* = 0.90 (supplemental Figure 11, available at http://links.lww.com/PAIN/A643). Heterogeneity was high (84.1%). Two additional prospective studies examined baseline alcohol abuse as a predictor of developing pain/neuropathy but could not be combined due to different analyses. Both studies reported a nonsignificant association between these variables.^60,77^ Seven cross-sectional studies provided events data or ORs. The pooled OR was not significant: OR = 1.22 (95% CI 0.92-1.62), *z* = 1.36, *P* = 0.17 (supplemental Figure 12, available at http://links.lww.com/PAIN/A643). Heterogeneity was medium (I^2^ = 39.0%).

### 3.7. Sleep disturbance

Three studies investigated sleep disturbance. Two cross-sectional studies reported mean values and SDs. The pooled effect was significant and showed moderately greater sleep problems in participants with vs without pain: SMD = 0.66 (95% CI 0.45-0.87), *z* = 6.12, *P* < 0.001. Heterogeneity was 0.0% (supplemental Figure 13, available at http://links.lww.com/PAIN/A643). Another cross-sectional (n = 45) study reported a significant correlation between pain severity and sleep disturbance, and a nonsignificant correlation between sleep and functioning.^94^

### 3.8. Antiretroviral nonadherence

Seven studies investigated the association between pain and suboptimal ART adherence. Data were analysed separately according to whether the adherence variable was coded in the direction of nonadherence or adherence. One prospective study (n = 258) reported that severe pain at baseline predicted higher odds (OR = 1.37, 95% CI 1.02-1.85) of follow-up ART nonadherence.^46^ One cross-sectional study provided events data, whereas another provided an OR. The pooled OR was significant and indicated that participants with pain were more likely to report nonadherence: OR = 1.40 (95% CI 1.07-1.82), *z* = 2.50, *P* = 0.01 (supplemental Figure 14, available at http://links.lww.com/PAIN/A643). Heterogeneity was 0.00%. One cross-sectional study (n = 42) found significant positive correlations between pain severity and adherence forgetfulness and fears.^57^

Two cross-sectional studies reported data for the association between pain and adherence (events data or OR). The pooled OR was less than one, indicating the likelihood of adherence was lower in participants with pain, but this was not statistically significant: OR = 0.32 (95% CI 0.08-1.32), *z* = 1.57, *P* = 0.12 (supplemental Figure 15, available at http://links.lww.com/PAIN/A643). Heterogeneity was high (74.3%). Finally, one low-quality cross-sectional study (n = 377) found that pain presence was not associated with adherence in a structural equation model.^72^

### 3.9. Health care use

Six studies examined health care use. One prospective study (n = 1521) found that baseline pain predicted significantly higher odds (OR = 1.6, 95% CI 1.2-2.0) of urgent care visits.^68^ Two cross-sectional studies reported events data. The pooled OR was not significant: OR = 0.98 (95% CI 0.58-1.66, *z* = 0.07, *P* = 0.94) (supplemental Figure 16, available at http://links.lww.com/PAIN/A643). Heterogeneity was 0.0%. Two further cross-sectional studies reported mean values and SDs. The pooled effect was small but significant, such that participants with pain had greater health care use than those without pain: SMD = 0.36 (95% CI 0.21-0.51, *z* = 4.66, *P* < 0.001). Heterogeneity was 0.0% (supplemental Figure 17, available at http://links.lww.com/PAIN/A643). One further cross-sectional study (n = 1120) found that participants with pain and daily opioid use had more clinic visits than those with pain without daily opioid use and those without pain (SD not reported).^53^

### 3.10. Missed HIV clinic visits

Two prospective studies reported ORs for baseline presence of pain predicting missed HIV clinic visits over 1-year follow-up. The pooled OR was significant, such that those with pain at baseline had higher odds of a missed HIV clinic visit: OR = 1.42 (95% CI 1.13-1.79), *z* = 2.98, *P* = 0.003 (supplemental Figure 18, available at http://links.lww.com/PAIN/A643). Heterogeneity was 0.0%.

### 3.11. Unemployment

Seven cross-sectional studies provided events data or ORs for the association between unemployment and pain. The pooled OR was significant and indicated that participants with pain had higher odds of being unemployed than those without pain: OR = 2.09 (95% CI 1.59-2.76, *z* = 5.25, *P* < 0.001) (supplemental Figure 19, available at http://links.lww.com/PAIN/A643). Heterogeneity was moderate (48.6%). One further cross-sectional study (n = 229) that did not have data available for meta-analysis likewise found that participants with pain were significantly more likely to be unemployed than those without pain.^79^

### 3.12. Protective factors

Five studies examined protective psychological factors. One prospective study (n = 62) found significant small and medium correlations between change in self-reported pain acceptance during CBT and posttreatment pain severity and interference, respectively.^86^ One cross-sectional case-control study observed lower resilience in participants with (n = 99) vs without pain (n = 98; medium effect); however, this study found nonsignificant correlations between resilience and pain severity and interference in the pain group.^121^

One high-quality cross-sectional study found that participants with pain (n = 170) reported lower disease management self-efficacy than did those without pain (n = 59) (small effect).^79^ Within the pain group in this study, there were nonsignificant correlations between self-efficacy and pain severity and interference, and a small positive correlation between self-efficacy and quality of life.^79^ One low-quality cross-sectional study found that those with greater adherence self-efficacy were less likely to report pain (n = 70).^8^ Finally, one low-quality cross-sectional study found lower mean self-reported optimism in participants with (n = 50) vs without pain (n = 46) (small effect).^101^

### 3.13. Social factors

Four studies investigated social factors. The BEACON study (n = 377) explored social processes across 3 papers, 2 of which describe prospective data (medium quality), whereas the third reported cross-sectional data (low quality). Baseline chronic pain predicted “negative social support” (ie, overly intrusive or insensitive responses from others and a lack of support) at 12 months, controlling for baseline social support.^73^ Another prospective analysis showed that no chronic pain at baseline predicted greater support reciprocity at follow-up.^74^ Chronic pain was associated with significantly poorer ratings of patient–provider engagement in cross-sectional analyses.^72^

Two studies examined self-reported stigma, but could not be combined. One medium-quality cross-sectional study (n = 50) found a moderate positive correlation between stigma and pain severity.^122^ One low-quality cross-sectional study (n = 201) found that participants with “pain disorder” reported higher stigma scores than those without “pain disorder.”^98^ One medium-quality cross-sectional study found no difference in mean number or quality of self-reported social supports between participants with (n = 274) and without pain (n = 164).^91^

## 4. Discussion

This review including over 13,000 participants found “some” or “moderate” evidence supporting an association between pain outcomes and depression, psychological distress, posttraumatic stress, drug abuse, sleep disturbance, health care use, missed HIV clinic visits, ART adherence, unemployment, and protective psychological factors in people with HIV. Surprisingly few studies have examined protective psychological factors or social processes. There is a lack of high-quality research on psychosocial factors related to chronic pain in people with HIV. These findings can inform future research and treatment development in this area.

The association between depression and poorer self-reported pain outcomes in HIV is consistent with the wider pain literature.^4,64^ Data from prospective studies suggest depression is a risk factor for pain. However, caution is warranted in this interpretation, given the observational nature of studies. There is likely a bidirectional relationship, with shared neurobiological pathways, cognitive appraisals, and behavioural disengagement underpinning this association.^4,7,14^ Evidence supporting the association between pain and sleep disturbance is consistent with the wider literature that reports reciprocal associations between pain, sleep, and depression.^106^

There was substantial variability in the assessment of “psychological distress,” which may have contributed to the statistical heterogeneity observed. Although different measures were used to assess variables such as pain catastrophizing, pain-related fear, stress, and general anxiety, these measures overlap conceptually and in item content. The consistency of results within the psychological distress category suggests the findings are robust across different assessment methods. Several studies assessed “mental illness” based on a range of diagnoses in participants' medical file without clear diagnostic criteria. Studies exploring mental health diagnoses should use valid and reliable criteria and, ideally, semi-structured clinician-administered interviews as the gold standard.^128^ In light of high rates of posttraumatic stress disorder (PTSD) in HIV,^99^ further research is particularly needed to understand the role of PTSD in pain in this context. Alternately, rather than focusing on specific mental health diagnoses, research investigating psychosocial processes that explain the impact of a range of psychological difficulties may prove useful moving forward.^42^

Few studies investigated fear-avoidance model variables, such as pain catastrophizing and pain-related fear, which have dominated the musculoskeletal pain literature. Fear of movement is strongly associated with musculoskeletal pain disability.^20^ However, neuropathic pain is often spontaneous and not clearly provoked by movement, although it may inhibit movement. Therefore, research is needed to determine the relevance of fear-avoidance model constructs in neuropathic pain that is common in HIV.

A bidirectional association between drug abuse and pain in HIV is suggested by prospective data showing opioid abuse as both predictor^60^ and outcome^114^ of pain. In a population where there are concerns about analgesic prescribing,^58^ poorly managed pain may contribute to increased abuse of nonprescribed opioids, which may be exacerbated by depression.^114^ Alternately, prolonged opioid abuse may disrupt descending pain inhibition, exacerbating pain.^49^ Differing definitions of drug and alcohol abuse across studies may account for variability in effects. Future research on substance abuse in this context should use validated assessments, either screening tools or diagnostic interviews that capture key features of abuse, such as continued use despite harm.^1,41,128^

Adherence to ART and retention in care are psychosocial factors unique to the HIV context and are of vital importance, given their associations with mortality, morbidity, and drug resistance.^112^ The finding that pain was associated with reduced ART adherence and missed HIV clinic visits highlights the necessity of adequate pain management in HIV. Understanding the links between pain, ART adherence, and retention in care likely requires consideration of other psychosocial factors, such as substance abuse and depression, which may mediate or moderate this association.^68,92^

Findings that pain was associated with greater unemployment and health care use highlight the individual, societal, and economic costs of pain in HIV. This is consistent with the broader literature, although health care use is typically underassessed in trials of psychotherapy for chronic pain.^87^ Studies assessing health care use were restricted to the United States, whereas the unemployment–pain link was consistent in studies from the United States, Russia, and South Africa. The association between pain and health care use differed across studies on the basis of the type of health care assessed. Assessment of the most frequently accessed services (eg, general practitioner visits), rather than relatively infrequent events (eg, hospitalisations), may increase the interpretability of future health care data.

Surprisingly, only 5 studies assessed protective psychological factors. The lack of studies on protective factors mirrors historical trends in the general pain literature, although there has been greater focus on protective factors more recently. The focus on “maladaptive” responses to pain is problematic because such responses can be understood as a function of their short-term utility.^126^ Moreover, abnormal conceptualizations often fail to specify pathways through which recovery and successful functioning occur when pain is present. The psychological flexibility model, within which pain acceptance has been conceptualised, might prove useful for future research.^63^

A recent proposal for updating the definition of pain highlights the central role of social factors.^127^ However, our review identified only 4 studies exploring interpersonal variables. The lack of research on stigma in relation to pain is particularly surprising because managing stigma is key to the success of the HIV/AIDS response.^108^ Stigma has recently been highlighted as important for the well-being of patients with chronic pain in general.^23,125^ Future research is needed to determine the function of stigma in chronic pain in people with and without HIV.

The study samples included in our review varied widely in terms of the proportion of men and women, participant age, ethnicity, and duration and severity of HIV. Our sensitivity analyses support the potential applicability of findings across pain types, ART treatment eras, and health care systems. Due to poor reporting of viral loads and CD4^+^ counts, our analysis stratifying by these indicators is difficult to interpret. Caution is also warranted regarding the cross-cultural applicability of the findings because most studies were from the United States. One South African study with a predominantly female sample found patients with and without pain did not differ on depression or anxiety, likely due to high scores across the sample.^88^ Socioeconomic factors, such as poverty and gender, may thus alter the relationships between pain, functioning, and mental health.^88,121^ Care is needed in applying Western psychological concepts in non-Western cultures.^51,82,83^ Research must also determine unique cultural features that influence the experience and expression of pain in HIV.

A guiding theoretical model is needed to integrate psychosocial processes relevant to pain and HIV. Such a model should make specific predictions about the relative contributions of cognitive, affective, behavioural, and sociocultural processes in relation to specific pain outcomes. This review identified a number of closely related psychosocial constructs. Therefore, a theoretical model may benefit from identifying a key set of nonoverlapping variables.^63^ This may draw on prominent models within the field of pain, such as the fear-avoidance^19^ and psychological flexibility models,^63^ and those within the HIV literature that focus heavily on sociocultural perspectives to understand the impact of processes, such as stigma, on well-being.^78^

The current findings suggest the relevance of psychosocial treatments to manage persistent pain in HIV. To the best of our knowledge, only 3 small RCTs have evaluated CBT and mindfulness-based treatment.^29,35,118^ Nonrandomized trials of CBT^113^ and hypnosis^25^ have also been conducted. Further evaluation of psychosocial treatments for HIV and chronic pain is thus needed. The development of treatments that specifically target psychosocial factors identified in this review with “some” or “moderate” evidence may prove fruitful.

Several limitations warrant consideration. We used a comprehensive search strategy that included efforts to identify gray literature to limit publication bias; however, relevant studies may have been missed, given the broad nature of the search. We used an adapted quality assessment tool. Although we based this on previously validated tools, the adaptations may have limited the reliability and validity of our quality assessment. Assessment of pain was inadequate in many studies. Future research should assess information regarding pain duration, intensity, location, and type. Studies investigating chronic pain should specify eligibility criteria in line with recognized definitions: the presence of daily, clinically meaningful pain intensity and functional interference for at least 3 months.^10,26,131^ Given the relevance of neuropathic pain in this population, the use of well-validated screening tools of neuropathy signs and symptoms is important.^16,33,129^

This review identified a large number of psychosocial factors. As evidence on specific psychosocial factors develops in this area, it may be useful for a future review to use a more targeted approach to synthesize data on a smaller number of prespecified variables. We focused on bivariate analyses and dichotomized multiple between-groups analyses to facilitate comparison across studies and minimise pairwise comparisons. However, this may have limited an in-depth understanding of psychosocial factors from multivariate models and more subtle subgroup analyses. Future research examining the association between psychosocial factors and pain outcomes in HIV should consider controlling for such variables as age, sex/gender, race/ethnicity, socioeconomic status, HIV duration, current and nadir CD4^+^ count and current and peak viral load, current and past ART regimens, and other medical comorbidities (eg, hepatitis C, diabetes, and tuberculosis). Where multiple psychosocial variables are included, sufficient rationale for each variable should be provided and care should be taken to minimize overlap in assessment content between variables.

Despite these limitations, this is the first systematic review to specifically explore psychosocial variables associated with persistent pain in HIV. From this review, it is recommended that researchers (1) focus greater attention on protective psychological factors and social processes, such as stigma and processes to undermine stigma; (2) use higher-quality assessment tools; and (3) develop and test treatments to target key psychosocial factors to improve pain outcomes in HIV. Improving quality of life is a priority as people with HIV live longer. Adequate, whole-person pain management is vital to achieve this goal.

## Conflict of interest statement

The authors have no conflict of interest to declare.

This research is an independent work supported by the National Institute for Health Research (NIHR Postdoctoral Fellowship, W. Scott, PDF-2015-08-059). L.M. McCracken is partly funded through the NIHR Biomedical Research Centre at South London and Maudsley NHS Foundation Trust and King's College London. The views expressed in this publication are those of the authors and not necessarily those of the NHS, the National Institute for Health Research, or the Department of Health. Dr. Kemp reports grants from the European Commission (FP7 Neuropain #HEALTH F2-2013-602891) during the conduct of the study. Dr. Rice reports grants from Orion Pharma, other from Spinifex/Novartis, personal fees from Imperial College Consultants, outside the submitted work. In addition, Dr. Rice has a patent, Rice A.S.C., Vandevoorde S. and Lambert D.M Methods using N-(2-propenyl)hexadecanamide and related amides to relieve pain. WO 2005/079771 pending, and a patent, Okuse K. et al., Methods of treating pain by inhibition of vgf activity EP13702262.0/ WO2013 110945, pending.

## Supplementary Material

SUPPLEMENTARY MATERIAL

## Appendix A. Supplemental digital content

Supplemental digital content associated with this article can be found online at http://links.lww.com/PAIN/A643.

## Supplemental video content

Video content associated with this article can be found at http://links.lww.com/PAIN/A645.
