# Supplementary material for: Psychosocial factors associated with persistent pain in people with HIV: a systematic review with meta-analysis
Source: Pain. 2018 Aug 16;159(12):2461–76. doi: 10.1097/j.pain.0000000000001369 (PMC6250281; doi:10.1097/j.pain.0000000000001369)
Supplement: SUPPLEMENTARY MATERIAL [file jop-159-2461-s001.docx]

Supplementary Table 1**.** Demographic characteristics and pain assessment of included studies.

| **Study** | **Age (years)**  **Mean (SD)** | **Sex (% men)** | **Race/Ethnicity (%)**  **(Pain/No Pain)** | **HIV Duration (y)**  **Mean (SD)** | **Pain/Function/QoL** |
| --- | --- | --- | --- | --- | --- |
| Aouizerat et al. (2010)[2] | Pain: 45.7 (8.1) No Pain: 44.4 (8.6) | Pain: 72.6 No Pain: 78.2 | White: 43.0/38.0 Black: 33.0/47.0 Hispanic: 11.0/9.0 | Pain: 12.5 (7.0) No Pain: 11.5 (6.9) | MSAS |
| Bakka (Thesis, 1995)[5] | Men: 40 (6.23) Women: 37 (7.10) | 84.1 | White: 48.4; Black: 31.0; Hispanic: 19.0 | Not Reported | BPI; KPS; MSAS |
| Banerjee et al. (2011)[6] | Pain: 51.6 (9.9) No Pain: 45.2 (9.8) | Pain: 85.70 No Pain: 72.9 | Not Reported | Not Reported | Neurological exam |
| Berg et al. (2009)[8] | 46.0 | 46.0 | Hispanic: 50.0 Black: 43.0 | Not Reported | BPI |
| Breitbart et al. (1996^1^; 1997; 1998); Rosenfeld et al. (1996)[11-13,91] | Pain: 39.0^1^  No Pain: 38.6 | Pain: 60.9^1^ No Pain: 68.9 | Black: 39.0/35.9^1^ White: 37.6/41.5  Hispanic: 23.4/22.6 | Pain: 4.25^1^  No Pain: 4.71 | BPI; FLIC; MSAS |
| Ellis et al. (2010)^1^ [27]  Keltner (2012)^2^ [48] | Pain: 46 (7.5)^1^ No Pain: 45 (8.0) | Pain: 74.9^1^ No Pain: 78.0 | White: 45.0^2^; Black: 43.0; Hispanic: 10.0 | Not Reported | Neurological exam; MOS-HIV |
| Evans et al. (1998)[28] | Pain: 42  No Pain: 40 | 100.0 | White: 59.0; Black: 19.0; Hispanic: 19.0 | Not Reported | VAS; EQOLESQ |
| Evans et al. (2003a^1^b^2^); Davis et al. (2004); Griswold et al. (2005)  [22,29,30,38] | 46.0 (7.9)^2^ | 70.6^2^ | Black: 49.4^1^  Hispanic: 18.8 White: 30.6 | Not Reported | BPI; KPS |
| Hansen et al. (2011)^1^  Miaskowski et al. (2011) Jeevanjee et al. (2014)  [39,46,70] | 49.5 (7.5)^1^ | Pain: 61.3^1^ No Pain: 95.5 | Black: 43.1/27.3^1^ White: 37.5/45.5 | Not Reported | BPI |
| Jiao et al. (2016) [47] | Pain: 50.0 (10.2) No Pain: 46.6 (11.5) | Pain: 50.0 No Pain: 62.0 | Black: 45.0/50.0  Hispanic: 44.0/36.0  White: 6.0/9.0 | Not Reported | ICD-9 diagnoses; NRS |
| Kirkland (Thesis; 2012)[50] | Pain: 43.28 (11.7)  No Pain: 43.2 (13.3) | Pain: 52.4 No Pain: 49.5 | Black: 74.8/73.1 | Pain: 8.6 (6.0)  No Pain: 8.0 (5.4) | Interview (IHS criteria); BHS; MIDAS; HIT-6 |
| Knowlton et al. (2015)^1^ Mitchell et al. (2016; 2017ab^2^) [52,72-74] | 48.15 (6.27)^1^ | 61.4^1^ | Black: 85.9^2^ White: 6.5 | Not Reported | Past 6 month pain |
| Koeppe et al. (2010) [53] | Pain: 45.61  No Pain: 42.2 | Pain: 81.5 No Pain: 85.1 | White: 71.2/64.6 | Pain: 10.53  No Pain: 7.9 | Medical file; NRS |
| Koeppe et al. (2012)[54] | 41.8 | 76.4 | White: 72.2 | 8.3 (5.3-12.3)† | Medical file; NRS |
| Lagana et al. (2002)[55] | 40.0 (7.5) | 57.5 | White: 57.5; Black: 31.7; Hispanic: 10.8 | 6.2 (3.5) | Past 6 month pain |
| Lopez et al. (2004)[56] | No AIDS: 36.8 (8.1)  AIDS: 39.5 (8.0) | 83.9 | White: 76.4 Non-White: 23.6 | Not Reported | Neurological exam |
| Lucey et al. (2011)[57] | 48 (7.4) | 85.0 | White: 50.0 Black: 48.0 | 5-10 years: 26.1% >10 years: 65.2% | NPS; BPI; PDI |
| Malvar et al. (2015)[60] | Pain: 44.0 (7.7) No Pain: 41.8 (8.8) | Pain: 76.0 No Pain: 83.0 | Black: 43.0/44.4 White: 47.0/41.0 Hispanic: 8.0/11.0 | Not Reported | Neurological exam |
| Mann et al. (2015)[61] | 50.3 (9.6) | 79.6 | Black: 34.3 White: 44.1 | <2 years: 15.7% >2 years: 84.3% | BPI; EQ-5D-3L; SF-12 |
| Merlin, Cen, et al. (2012)[65] | 47.5 (21-71) | 72.3 | Black: 58.4 | 11 (0-25)† | BPI; MSAS |
| Merlin, Westfall, et al. (2012) [68] | 43.7 (36.0–50.0) | 77.5 | Non-white: 52.3 White: 47.7 | Not Reported | EuroQoL |
| Merlin et al. (2015; 2017^1^)[66,67] | >50: 52.9%^1^ | Pain: 68.6^1^ No Pain: 74.3 | Black: 57.1/54.3^1^  White: 41.4/44.3 | Not Reported | BCPQ; PEG |
| Morgello et al. (2004)[76]  Fellows et al. (2012)^1^ [31] | Pain: 46.6 (7.1)^1^ No Pain: 43.7 (7.5) | Pain: 70.0^1^ No Pain: 58.6 | Black: 51.3/49.6^1^ Hispanic: 21.3/31.3 White: 27.3/18.6 | Pain: 11.9 (5.2) ^1^ No Pain: 11.2 (5.2) | Neurological exam |
| Nakamoto et al. (2010)[77] | 44.2 (10.2) | 84.0 | White: 51.0 | 10.4 (6.3) | Neurological exam |
| Parker et al. (2017) [79] | 30.7 (4.8) | Women:  100.0 | amaXhosa: 100.0 | Pain: 4.3 (3.3)  No Pain: 3.8 (3.2) | BPI; EQ-5D |
| Passik et al. (2006)[81] | 39.71 (6.59) | 86.0 | White: 45.0 Black: 52.0 | Not Reported | BPI; MSAS |
| Phillips et al. (2014)[84] | Pain: 51.3 (8.4) No Pain: 47.7 (8.9) | Pain: 89.3 No Pain: 84.2 | White: 85.7/86.8 African: 10.7/10.5 Asian: 0/2.6 | Pain: 17.8 (7.0) No Pain:14.7 (7.8) | BPNS, TCSS, UENS; NPSI; BPI; SF-36; |
| Pierson (Thesis; 2009)^1^ [86]  Cucciare et al. (2009)[21] Huggins et al. (2012)[44] Trafton et al. (2012)[113] | 49 (8)^1^ | 62.9^1^ | Black: 43.5^1^ White: 33.9 Hispanic: 11.3 Asian/Native: 4.8 | Not Reported | POQ |
| Pillay et al. (2017)[88] | Pain: 42.4 (11.3) No Pain: 44.6 (13.4) | Pain: 25.0 No Pain: 24.0 | Black: 100.0 | Pain: 9 (5–12) No Pain: 12 (10–15)† | BPNS; NRS; WBPQ; EQ5D |
| Robbins et al. (2013^1^; 2016) [89,90] | Pain: 43.5 (1.2) ^1^ No Pain: 41.4 (0.6) | Pain: 32.1^1^ No Pain: 48.5 | Thai: 95.7^1^ | Pain: 10.5 (0.8) ^1^  No Pain: 9.1 (0.4) | BPI; S-LANNS |
| Safo et al. (2017)[92] | 42 (32–49) | Women: 100.0 | Hispanic: 34.6/23.9 Black: 60.0/69.1 | Not Reported | CDC Healthy Days |
| Sandoval et al. (2014)[94] | 48.42 (8.13) | 58.0 | Black: 53.0; White: 33.0; Hispanic: 11.0 | 10.8 (6.8) | NPS; Physical performance |
| Saylor et al. (2017)[95] | 35 (8) | 53.0 | Black: 100.0 | Not Reported | TNS; PAOFI, KPS; |
| Schifitto et al. (2002)[96] | Pain: 40.8 (7.3) No Pain: 40.4 (7.6) | Pain: 80.0 No Pain: 81.5 | White: 52.7/44.4 | Not Reported | Neurological exam; MOS; KPS |
| Schifitto et al. (2005)[97] | Pain: 43.6 (6.9) No Pain: 42.7 (7.0) | Pain: 70.7 No Pain: 75.7 | White: 32.6/11.7  Black: 61.0/77.5  Hispanic: 4.3/9.0 | Pain: 8.2 (4.0) No Pain: 7.2 (4.5) | Neurological exam; NPS; MOS; KPS |
| Shacham et al. (2015)[98] | 43.06 (11.05) | 68.7 | ‘Minority’: 72.6  White: 27.4 | Not Reported | DIS-IV |
| Simmonds et al. (2005)[101] | 40.70 (7.49) | 78 | White: 29.0; Black: 54.0; Hispanic: 13.0 | 6.77 (4.2) | NRS; MOS-HIV; Performance |
| Simms et al. (1992)[102] | Pain: 37.5 (7.6)  No Pain: 37.0 (6.5) | Pain: 46.0 No Pain: 77.0 | Not Reported | Pain: 2.81 (1.2) No Pain: 1.8 (1.2) | Medical exam |
| Singer et al. (1993; 1996^1^)[104,105] | Pain: 38.1 (9.7) ^1^ No Pain: 39.9 (10.6) | 100.0^1^ | ‘Predominantly Caucasian'^1^ | Not Reported | IHS criteria; KPS |
| Smith et al. (2002)[107] | PTSD: 41.47 (6.8)  No PTSD: 42.9 (7.8) | PTSD: 59.7  No PTSD: 57.8 | PTSD/No PTSD: Black: 37.1/62.9 Hispanic: 67.5/32.5 | Not Reported | BPI; SF-12 |
| Surratt et al. (2015)[110] | Pain: 46.9 (7.7)  No Pain : 45.3 (7.9) | Pain: 55.4 No pain: 63.7 | Black: 66.7/69.0 | Pain:13.6 (7.3) No pain:12.9 (7.2) | GAIN Health Distress |
| Tsui et al. (2012; 2016^1^)[116,117] | Pain: 43.5(7.3)^1^ No Pain: 41.9 (7.5) | Pain: 73.4^1^ No Pain: 75.6 | Black: 39.2/43.0^1^ White: 36.1/30.6 Hispanic: 17.7/19.4 | Not Reported | SF-12; HIV Symptom Index |
| Tsui et al. (2013; 2014)[114,115] | Pain : 30.4 (5.5) No Pain : 29.8 (5) | Pain: 53.0 No Pain: 65.0 | Not Reported | Pain: 4.6 (3.6) No Pain: 4.2 (3.5) | SF-12 pain interference |
| Uebelacker et al. (2015)[119] | Pain: 51.0 (8.4) No Pain: 51.0 (10.3) | Pain: 58.0 No Pain: 68.0 | White: 68.0/62.0  Black: 28.0/31.0 | Pain:17.9 (9.5) No Pain:16.0 (7.9) | Past 6 month pain; NRS; BPI |
| Wadley et al. (2016)[121] | Pain: 44 (10)  No Pain: 40 (10) | Pain: 34.0 No Pain: 22.0 | Black: 100.0 | Pain: 6 (1–25)†  No Pain: 6 (1–20) | BPI; EQ5D3L |
| Wadley et al. (Unpublished)[122] | 45 (10) | 22.0 | Not Reported | 8 (5) | NRS |

**Note:** BCPQ, Brief Chronic Pain Questionnaire; BHS, Brief Headache Screen; BPI, Brief Pain Inventory; BPNS, Brief Peripheral Neuropathy Screen; CDC, Centre for Disease Control; DIS-IV, Diagnostic Interview Schedule for DSM-IV; EQOLESQ, Endicott Quality of Life Enjoyment and Satisfaction Questionnaire; FLIC, Functional Living Index; GAIN, Global Appraisal of Individual Needs; HIT-6, Headache Impact Test; KPS, Karnofsky Performance Scale; IHS, International Headache Society; MIDAS, Migraine Disability Assessment; MOS-HIV**,** Medical Outcomes Study HIV Health Survey; MSAS, Memorial Symptom Assessment Scale**;** NPS, Neuropathic Pain Scale; NPSI, Neuropathic Pain Symptom Inventory; NRS, Pain Intensity Numerical Rating Scale; PAOFI, Patient Assessment of Own Functioning Inventory; PDI, Pain Disability Index; POQ, Pain Outcomes Questionnaire; PTSD, Post-traumatic stress disorder; SF-12/36, Short form Medical Outcome Survey; S-LANNS, Self-Administered Leeds Assessment of Neuropathic Symptoms and Signs; TCSS, Toronto Clinical Scoring System; TNS, Total Neuropathy Scale; UENS, Utah Early Neuropathy Scale; VAS, Pain intensity Visual Analogue Scale; WBPQ, Wisconsin Brief Pain Questionnaire

^†^Median and Range
