# Supplementary material for: Psychosocial factors associated with persistent pain in people with HIV: a systematic review with meta-analysis
Source: Pain. 2018 Aug 16;159(12):2461–76. doi: 10.1097/j.pain.0000000000001369 (PMC6250281; doi:10.1097/j.pain.0000000000001369)
Supplement: SUPPLEMENTARY MATERIAL [file jop-159-2461-s002.docx]

**Multi-OMICS Study of Frailty and its Genetic Correlation with Common Widespread Pain.** Gregory Livshits, Ida Malkin, Ruth Bowyer, Serena Verdi, Jordana Bell, Cristina Menni, Frances M. K. Williams**,** Claire J. Steves

**Supplementary material.**

**Fig S1.** Manhattan plot of GWAS results for metabolites highly associated with FI-scores, and FI score itself

**
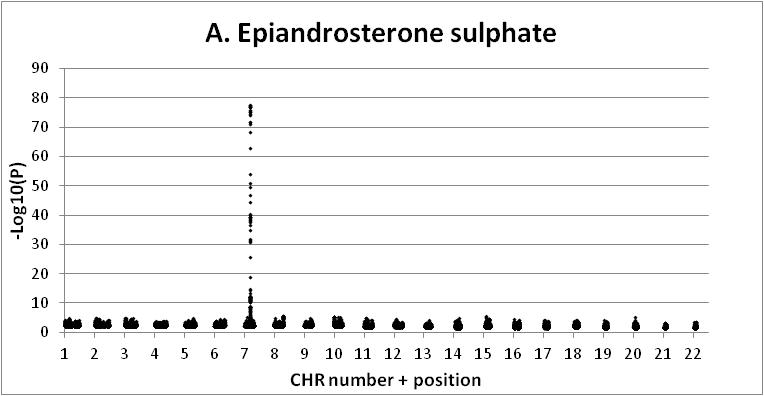
**

**
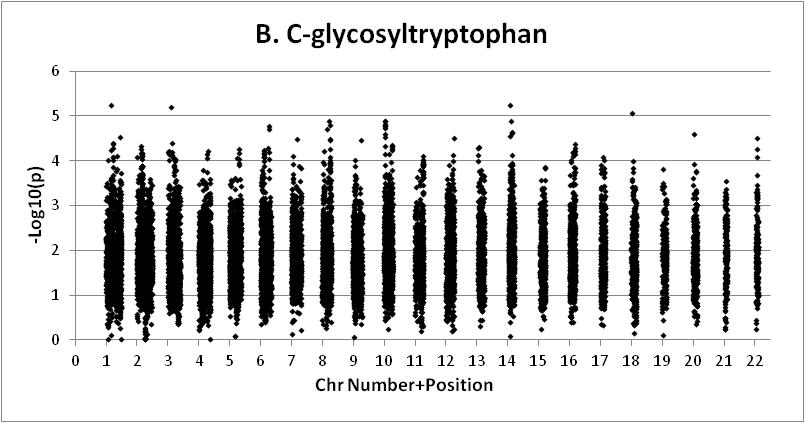
**

**
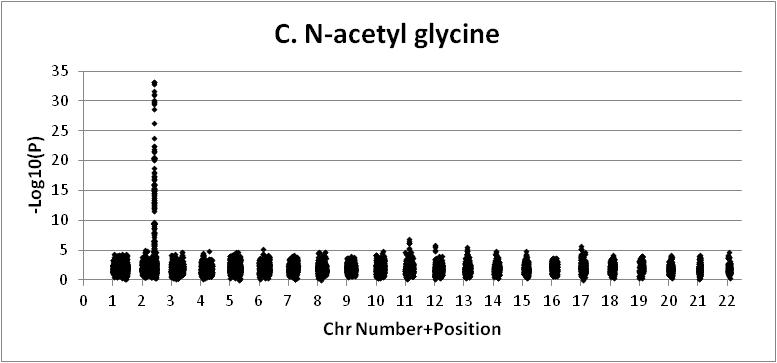
**

**
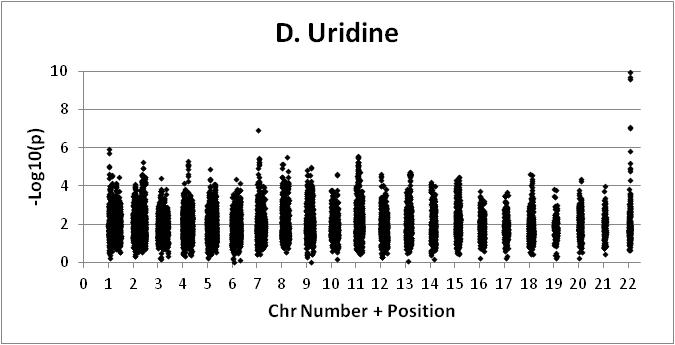
**

**
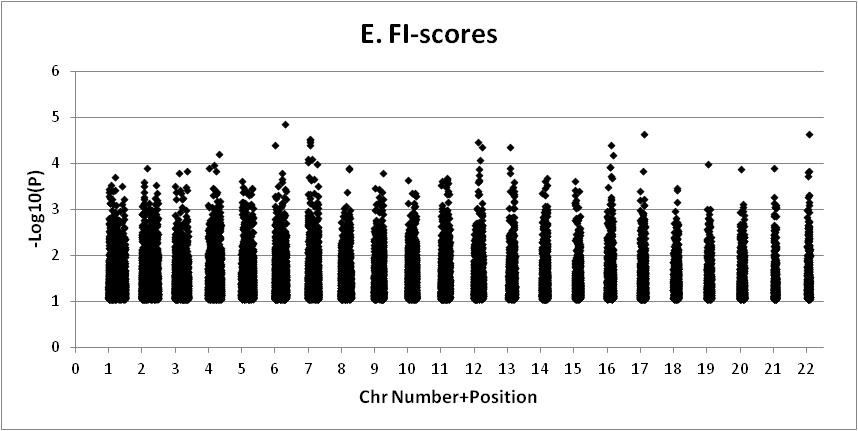
**
